# Lower HIV Provirus Levels Are Associated with More APOBEC3G Protein in Blood Resting Memory CD4+ T Lymphocytes of Controllers *In Vivo*

**DOI:** 10.1371/journal.pone.0076002

**Published:** 2013-10-16

**Authors:** MariaPia De Pasquale, Yordanka Kourteva, Tara Allos, Richard T. D'Aquila

**Affiliations:** 1 Division of Infectious Diseases, Department of Medicine, Vanderbilt University School of Medicine, Nashville, Tennessee, United States of America; 2 Division of Infectious Diseases and Northwestern HIV Translational Research Center, Department of Medicine, Northwestern University Feinberg School of Medicine, Chicago, Illinois, United States of America; New York University, United States of America

## Abstract

Immunodeficiency does not progress for prolonged periods in some HLA B57- and/or B27-positive subjects with human immunodeficiency virus type 1 (HIV) infection, even in the absence of antiretroviral therapy (ART). These “controllers” have fewer HIV provirus-containing peripheral blood mononuclear cells than “non-controller” subjects, but lymphocytes that harbor latent proviruses were not specifically examined in studies to date. Provirus levels in resting memory cells that can serve as latent reservoirs of HIV in blood were compared here between controllers and ART-suppressed non-controllers. APOBEC3G (A3G), a cellular factor that blocks provirus formation at multiple steps if not antagonized by HIV virion infectivity factor (Vif), was also studied. HLA-linked HIV control was associated with less provirus and more A3G protein in resting CD4+ T central memory (Tcm) and effector memory (Tem) lymphocytes (provirus: p = 0.01 for Tcm and p = 0.02 for Tem; A3G: p = 0.02 for Tcm and p = 0.02 for Tem). Resting memory T cells with the highest A3G protein levels (>0.5 RLU per unit of actin) had the lowest levels of provirus (<1,000 copies of DNA per million cells) *in vivo* (p = 0.03, Fisher's exact test). Using two different experimental approaches, Vif-positive viruses with more A3G were found to have decreased virion infectivity *ex vivo*. These results raise the hypothesis that HIV control is associated with increased cellular A3G that may be packaged into Vif-positive virions to add that mode of inhibition of provirus formation to previously described adaptive immune mechanisms for HIV control.

## Introduction

The small proportion of human immunodeficiency virus type 1 (HIV-1)–infected individuals who spontaneously control HIV-1 in the absence of antiretroviral therapy (ART) may provide insights to help forestall pathogenesis in non-controllers. Until their control is lost, ‘elite controllers’ (EC) maintain undetectable levels of viremia, while ‘viremic controllers’ (VC) have plasma HIV RNA consistently <2000 copies/mL. Recovery of HIV-1 *ex vivo* is also decreased from controllers' CD8+ T cell-depleted blood cells [1], [2], and this is likely due to the lower level of integrated HIV provirus found in controllers' peripheral blood mononuclear cells (PBMC) [2]. Although controllers have consistently been found to have lower provirus levels than untreated non-controllers [2]–[5], two studies comparing PBMC provirus levels in controllers versus non-controllers with viremia well-controlled by ART, called ART–suppressed (AS) non-controllers, had conflicting results. One report identified lower provirus levels in controllers [4], but a second did not [2]. This raised a question about whether decreased provirus is a consistent, distinguishing feature of spontaneous control. We now extend provirus quantitation from PBMC to resting CD4+ T memory lymphocyte subtypes, including the T central memory (Tcm) cells that specifically harbor quiescent proviruses constituting HIV-1's latent reservoir in blood [6]. We find decreased provirus levels in those cells from controllers, compared to cells from AS non-controllers.

Understanding mechanisms that cause this decrease in provirus levels in controllers' cells may help develop a vaccine or a strategy for functional cure of HIV. Most, but not all, controllers have HLA B57 and/or 27 alleles that allow CD8+ cytotoxic T lymphocytes (CTL) to recognize conserved HIV capsid (CA) epitopes [7]–[10]. The lack of viremia rebound when CTL escape mutants emerge, and the fact that some controllers do not have CTL activity directed against conserved epitopes, indicates this is not the only mechanism underlying the complex long-term phenotype of lower viremia, slower loss of CD4+ T cells and decreased rate of progression to immunodeficiency. Additional mechanisms have been hypothesized to interact with CTL killing of HIV-infected cells in causing a broadly interconnected and durable defense against HIV pathogenesis [2]–[4], [7]–[15]. We have been studying the possible contribution of an intrinsic cellular restriction factor, Apolipoprotein B mRNA-editing enzyme catalytic, polypeptide-like 3G (APOBEC3G, abbreviated A3G here), to control either independently or by augmenting CTL-based mechanisms.

In an earlier report, we added evidence to the literature that A3G provides anti-HIV-1 activity *in vivo* [5]. In that report, peripheral blood mononuclear cells (PBMC) from controllers, which had lower provirus burden than cells from untreated non-controllers, were shown to have higher A3G mRNA levels [5]. In the current study, those earlier analyses were extended and inverse associations were identified between A3G protein and provirus levels in HLA B57- and/or 27-positive controllers' resting CD4+ T memory lymphocytes *in vivo*. Virus was also produced *ex vivo* in the current experiments from cells with different levels of cellular A3G to determine if increased cellular A3G could gain access to virions and affect their infectivity. Results suggested that some of the relatively increased cellular A3G escaped degradation by the HIV virion infectivity factor (Vif), and was packaged into virions. These virions had decreased infectivity. The hypothesis that increased A3G adds to multiple, inter-locking mechanisms of durable HIV control is consistent with, and extends, earlier reports [2]–[5], [11]–[15].

## Methods

### Ethics Statement

All participants provided written informed consent under a protocol approved by the Health Sciences Committee 1 Institutional Review Board of the Vanderbilt University Human Research Protection Program.

### Study Subjects

Eleven AS non-controller subjects were recruited from the Comprehensive Care Center in Nashville, TN. The Vanderbilt Meharry CFAR HIV Immunopathogenesis Core provided cryopreserved PBMC from seven VC subjects in the cohort assembled by Dr. Kalams. VC subjects were infected for over 7 years with steady state plasma viral load <2,000 copies/ml at every measurement during the 5 years prior to the specimen studied here. Each VC subject had HLA B57 and/or B27 alleles associated with HIV-1 control [7], [8]. Median (IQR) plasma viral load for VC and AS subjects were respectively 94 (50;253) and 50 (48;50) copies/ml, (Mann Whitney test, p = 0.07). Median (IQR) CD4 counts for VC and AS subjects were 661 (360; 841) and 529 (215;935)/ml, respectively (Mann Whitney test, p = 0.4). When all 18 subjects were grouped together (including both AS non-controller and VC subjects), median viral load was 57 copies/ml (range 48–1000) and median CD4 cell count was 707 cells/ml (range 192-1161).

### Cells

PBMC were isolated from human blood by Ficoll-Paque (Amersham Biosciences) density gradient centrifugation. CD4+ T cells were negatively selected using magnetic beads to exclude cells positive for CD8, CD14, CD16, CD19, CD20, CD36, CD56, CD123, TCRg??d, and Glycophorin A (RoboSep, Stem Cell Technologies, Vancouver, Canada). CD4+ T cell subsets were further purified by four way sorting via 7-color flow cytometry (FACS Aria, BD) using labeled antibodies for CD4, CCR7, CD45RO, and the four activation markers CD25, CD69, CD38 and HLA-DR. The activated cells (positive for all 4 activation markers) were separated from resting cells (negative for all 4 activation markers), including resting CD4+ T central memory (Tcm: CCR7+, CD45RO+), resting CD4+ T effector memory (Tem: CCR7-, CD45RO+) and resting CD4+ T naïve (Tn: CCR7+, CD45RO-) cell subsets. The cells identified as Tem here include those called both Ttm and Tem by Chomont, et al. [6]. Briefly, CD4+ cells were incubated for 20 min at 37°C with a APC-anti-CCR7 (R&D Systems) followed by 20 minutes at room temperature with a combination of the other antibodies, including FITC-anti-CD45RO, PE TR-anti-CD4, PE-anti-CD69, PE-anti-CD38, PE-anti-CD25 and PE-anti HLA-DR (BD Pharmingen, CA), washed and sorted.

PBMC were cultured in R10 medium, consisting of RPMI 1640 media supplemented with 10% FBS, 100 U/ml penicillin G, 100 U/ml streptomycin, 2 mM glutamine, 50 U/ml IL-2 (AIDS Research and Reference Reagent Program, NIAID, NIH). Memory cell subtype culture used R10 supplemented with 10× MEM Vitamins solution, 10× MEM Non essential amino acids solution, and 10 mM Sodium Pyruvate (Cellgro Mediatech, VA). CD4+ T cells and their sorted subsets were activated in CD4+ medium with anti-CD3/CD28 antibody coated beads (Dynal/Invitrogen, Carlsbad, CA) for 7–10 days.

### Quantification of HIV-1 provirus

Chromosomal DNA was separated from unintegrated HIV-1 DNA in the presence of 1 M NaCl and 0.6% SDS. This was followed by further purification of cellular genomic DNA with Genomic DNA Purification Kit (Gentra Systems, Minneapolis, MN). Integrated HIV-1 provirus was then quantified using a modified 2-step *Alu*-PCR assay as previously described [5]. ACH-2 cells (NIH AIDS Research and Reference Reagent Program, Division of AIDS, NIAID, NIH) were used as an HIV-1 DNA copy number standard in a constant DNA background. The coefficient of variation (CV) between replicates was <50%.

### RNA isolation and real-time PCR (qRT-PCR)

RNA was isolated from cells using RNeasy kit (Qiagen, Valencia, CA) and quantified with RiboGreen RNA Quantitation Kit (Molecular Probes, Eugene, OR). After normalization by RNA concentration, A3G transcripts were quantified by TaqMan qRT-PCR (Applied Biosystems Prism 7000 Sequence Detection System, Foster City, CA) using probes and primers described previously [16]. Values are expressed as copies of transcripts normalized by 18 S and total RNA amount.

### Immuno-blotting and protein quantification

Quantitative immune-blotting using Li-cor Odyssey system (Li-Cor Biosciences, Lincoln, NE) assessed cellular A3G protein levels. Cells were lysed in 50 mM HEPES, pH 7.4, 60 mM NaCl, 0.2% NP-40, 0.1 mM PMSF and EDTA-free protease inhibitor cocktail (CalBiochem, San Diego, CA). Protein concentrations were determined using the Bradford protein assay reagent (Bio-Rad, Hercules, CA Lysates were normalized by protein content and 1 ug was separated on a SDS-PAGE followed by transfer to nitrocellulose membranes (Bio-Rad, Hercules, CA) for quantitative detection using the Li-Cor Odyssey system (Li-Cor Biosciences, Lincoln, NE). Target A3G proteins were probed with primary polyclonal antibody for A3G (anti-ApoC17 from Dr. K. Strebel through the NIH AIDS Research and Reference Reagent Program) and detected with a goat anti-rabbit secondary antibody conjugated to IRDye 800 (Li-Cor Biosciences). Subsequently, the membranes were probed for beta-actin as a loading control using monoclonal antibody clone AC-74 (Sigma, St. Louis, MO) followed by a donkey anti-mouse IRDye 680 (Li-Cor Biosciences). Infrared-fluorescent signals were quantified by the Li-Cor Odyssey system and results expressed as relative light intensity (RLU) of A3G per RLU of beta-actin.

For quantification of virion-packaged A3G, virus supernatants were harvested from cell culture and diluted 1∶2 with PBS. Briefly, 50 ml of washed Viro-Adembeads (Ademtech, France) were added to supernatants to capture and concentrate virions. ViroAdembeads kit is based on biomagnetic separation technology. After 20 min of agitation to room temperature, bead-virus complexes were collected with a magnet, washed, resuspended in PBS and loaded in a 96 well plate (Corning). 2 ng of p24- equivalents of HIV were loaded per well. Virions were fixed with 2% Paraformaldehyde and permeabilized with 0.05% TritonX/PBS for 10 min at room temperature and blocked for 1 hr with Odyssey Blocking buffer (Li-Cor Biosciences). Bead-virus complexes were kept attached to the bottom of the plate by a magnetic platform. A3G was probed over night at 4°C with mA3G antibody (obtained through the NIH AIDS Research and Reference Reagent Program, Division of AIDS, NAID NIH) and anti- p17 pAb for normalization. Then the plates were washed in 0.1%Tween-20/PBS, incubated for 1 h with secondary detection antibodies (IRDye 800 CW-conjugated anti-mouse and IRDye 680 CS-conjugated anti-rabbit, Li-Cor Biosciences), washed in 0.1%Tween-20/PBS and scanned for infrared signal using the Odyssey Imaging System (Li-Cor Biosciences).

### Virus production

Infections of healthy HIV-negative subjects' CD4+ T cell subsets were performed using the Vif-positive HIV-1 clinical isolate #14. Prior to infection, virus stocks were treated with 60 U DNAse I (Bio Rad) for 1 hr at 37°C. Three days after activation with anti-CD3/CD28 antibody-coated beads (Invitrogen), each cell type was infected with 0.01 MOI of virus stock for 3 hrs, then washed and resuspended in CD4+ T cell medium.

Endogenous HIV-1 was recovered from infected subjects' activated T cells by stimulating cells with 1 mg/ml anti-CD3:anti-CD8 bispecific antibody (gift of Dr Johnson Wong, Harvard Medical School, Boston MA) [17], which activates CD4+ T cells and depletes cytotoxic CD8+ T cells, in presence of IL2.

No allogeneic cells were added to cultures of either uninfected or infected subject cells. Virus production was quantified by p24 antigen ELISA of cell-free culture supernatants.

### Infectivity assay

After determination of the concentration of viral particles by p24 ELISA, 100 pg of p24-equivalents of HIV-1 were used to infect the TZM-bl indicator cells (AIDS Research and Reference Reagent Program, NIAID, NIH) cultured in DMEM with 10% FBS [18]. Luciferase activity was determined in cell lysates 72 hours after infection (Bright-Glo Luciferase assay substrate, Promega, Madison, WI; TopCount scintillation counter, Packard/Perkin Elmer, Waltham, MA). Data are shown as RLU per picograms of p24 added.

### Statistical Analysis

Means were compared using two-tailed Mann-Whitney test. P values of less than 0.05 were deemed statistically significant. Correlations were assessed by Spearman's rank correlation coefficients. Association between variables was evaluated by 2-tailed Fisher's exact test. Calculations were performed with the GraphPad Prism software (GraphPad Software, CA).

## Results

### Provirus and A3G levels in resting CD4+ T lymphocyte subtypes *in vivo*

**Figure 1:**
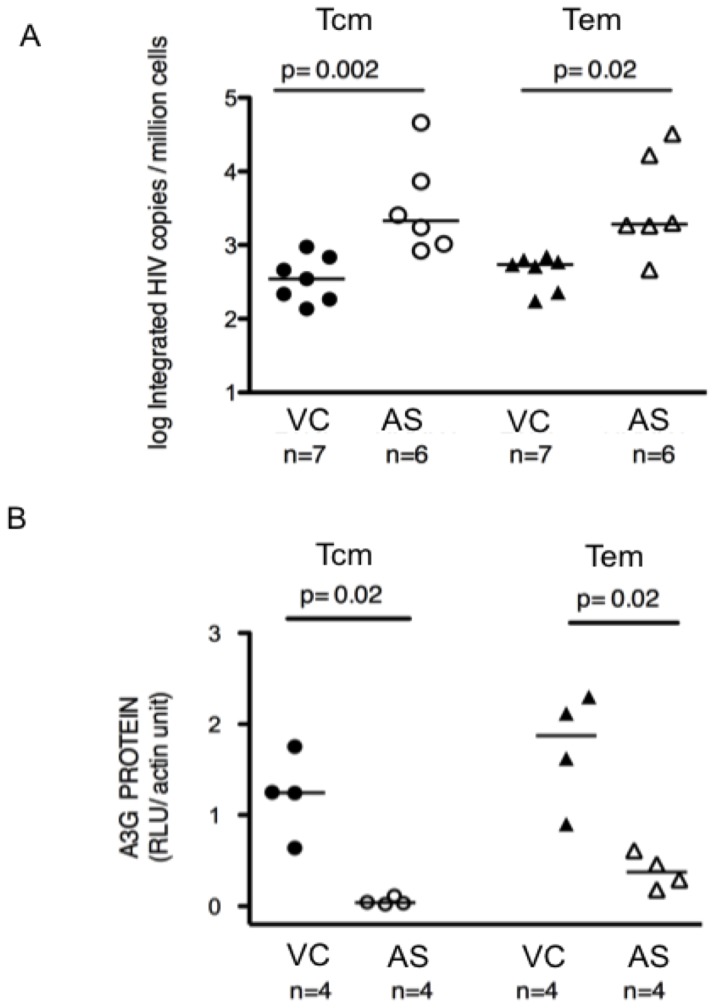
HIV provirus (A) and APOBEC3G protein (B) levels in blood resting CD4+ T memory lymphocytes. (**A**) Blood resting CD4+ T central memory (Tcm) and effector memory (Tem) cells from viremic controller (VC) subjects (n = 7) each had lower HIV provirus copy numbers than those cells from antiretroviral therapy suppressed (AS) non-controller subjects (n = 6). *Alu*-PCR was used to determine HIV provirus copy numbers, as log integrated copies per million cells. (**B**) Blood resting CD4+ Tcm and Tem cells from VC subjects (n = 4) each had higher APOBEC3G (A3G) protein levels than those cells from AS non-controller subjects (n = 4). Relative light units (RLU) of A3G bands on immunoblots were quantified using Licor Odyssey, with normalization to β-actin. Cells from which A3G protein levels were determined were from 8 of the same time points studied for each subject in Fig. 1(A) (4 for VC and 4 for AS subjects); protein quantitation was possible for only a subset of the subject cells/time points studied in Fig. 1(A). In (**A**) and (**B**), lines indicate median values. P values were determined by 2-tailed Mann-Whitney test.

We evaluated levels of HIV-1 provirus and A3G protein, as well as A3G RNA, in resting CD4+ T lymphocytes from blood of VC and AS non-controller subjects. All subjects had low viral load, as well as high CD4 cell counts (see Study Subjects in Methods). The AS non-controllers had lower viremia levels than the VC subjects (<50 copies/ml for AS non-controllers versus <2,000 copies/ml for VC subjects). Provirus levels determined by *Alu*-PCR were significantly lower in resting CD4+ T central memory (Tcm) cells from VC than from AS subjects (Fig. 1A, p = 0.002, Mann Whitney). Similarly, resting CD4+ T effector memory (Tem) cells from VC had fewer proviruses than those from AS subjects (Fig. 1A, p = 0.02, Mann Whitney).

We next assessed A3G protein levels in these cells. A3G protein levels were higher in each of the resting Tcm and Tem cells from VC subjects than in those cells from AS subjects (p = 0.02 for Tcm and p = 0.02 for Tem, respectively, Mann Whitney) (Fig. 1B). All Tcm and Tem cells from VC subjects had A3G protein levels quantified as >0.5 relative light units (RLU) per unit of actin, while only a single AS subject's Tem cells had A3G protein levels >0.5 RLU per unit of actin (p = 0.001, Fisher's exact test). There was also a significant association between A3G protein levels and provirus level when evaluated across all subjects' cells. Resting memory T cells with A3G protein levels >0.5 RLU per unit of actin were more likely to have <1,000 copies of provirus DNA per million cells (8 of 9 subjects' cells with A3G >0.5 RLU, versus 2 out of 7 subjects' cells with A3G <0.5 RLU per unit of actin; p = 0.03, Fisher's exact test).

**Figure 2:**
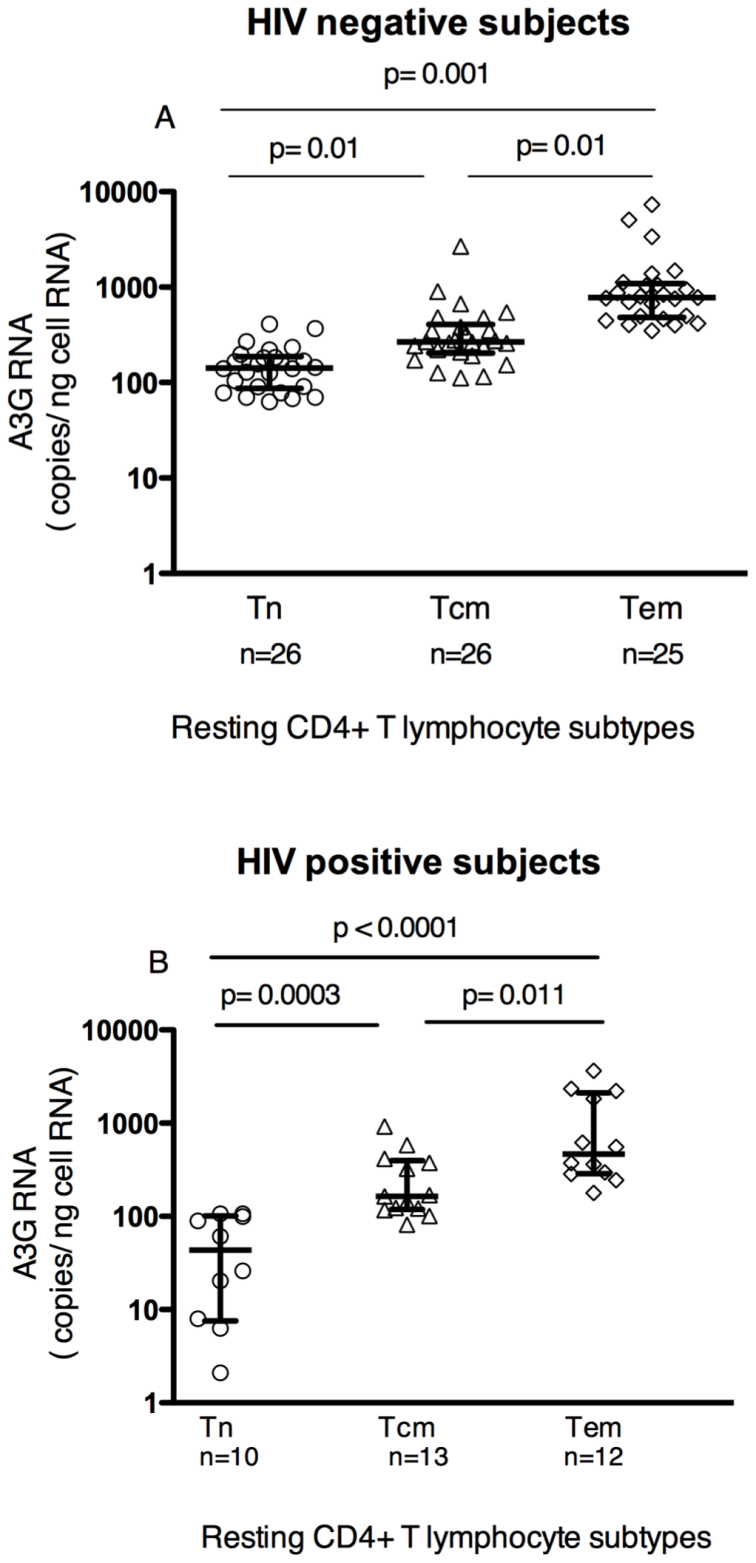
APOBEC3G RNA levels in blood resting CD4+ T memory lymphocyte subtypes. A3G RNA levels, determined by qRT-PCR with normalization to total cell RNA, progressively increased with differentiation state of resting CD4+ T memory lymphocytes, with naïve (Tn) < central memory (Tcm) < effector memory (Tem) lymphocytes. The same pattern was seen in these cells from HIV negative (**A**) and HIV positive (**B**) subjects. In (**A**) and (**B**), lines in plots represent median and inter-quartile range (IQR) values. P values were determined by 2-tailed Mann-Whitney U test.

We also compared A3G RNA levels among the different resting memory T cell subtypes. A3G RNA levels progressively increased with stage of differentiation from Tn to Tcm to Tem in cells from both uninfected and infected subjects (Fig. 2A and B). The increasing A3G RNA level with resting memory T cell differentiation was also seen in an analysis limited to the HIV-infected VC subjects (Fig. S1). VC subjects' Tem cells had A3G levels that did not differ from those of activated T cells (Fig. S1). There was no difference in A3G RNA levels in resting naïve (Tn), Tcm, or Tem cells from the chronically infected subjects studied above (all infected subjects treated as a single group) when compared to cells from uninfected subjects, indicating that chronic Vif-positive HIV-1 infection *in vivo* was not associated with any change in A3G RNA levels.

Since cellular activation precedes each successive stage of T cell differentiation, we directly tested if activating T cell receptors (TCR) on total CD4+ T lymphocytes from HIV-1 infected subjects increased A3G RNA, as reported earlier for total CD4+ T lymphocytes from uninfected subjects [19]–[21]. After *ex vivo* exposure of total CD4+ T cells from HIV-1 infected subjects to anti-CD3/CD28 beads (Dynal/Invitrogen, Carlsbad, CA), A3G RNA and protein levels both increased (Fig. S2).

We also considered another possible explanation for increased A3G levels seen in controllers' PBMCs in the prior study [5]. Given that A3G RNA increases with cell activation, we evaluated whether a higher percentage of activated cells were present in total CD4+ T cells from controllers than AS non-controllers. The percentages of activated cells (positive for HLA-DR, CD25, CD69 and CD38) were higher in CD4+ T cells from AS non-controller subjects than from either VC or HIV-negative control subjects (p = 0.005 and 0.0022, respectively; Fig. S3A). This agrees with earlier reports [22]–[26] and is not consistent with the possibility that higher A3G RNA in controllers' PBMCs noted previously was due to a higher proportion of activated cells in PBMC from controllers [5]. In addition, the proportions of resting Tcm and Tem cells did not differ between VC subjects and AS non-controllers (Fig. S3B). Differences in proportions of resting cells between controllers and non-controllers, therefore, were not likely to account for the results in PBMC in the prior study [5].

### Increased A3G and decreased infectivity of endogenous HIV-1 virions released from *in vivo* activated T cells during *ex vivo* culture

**Figure 3:**
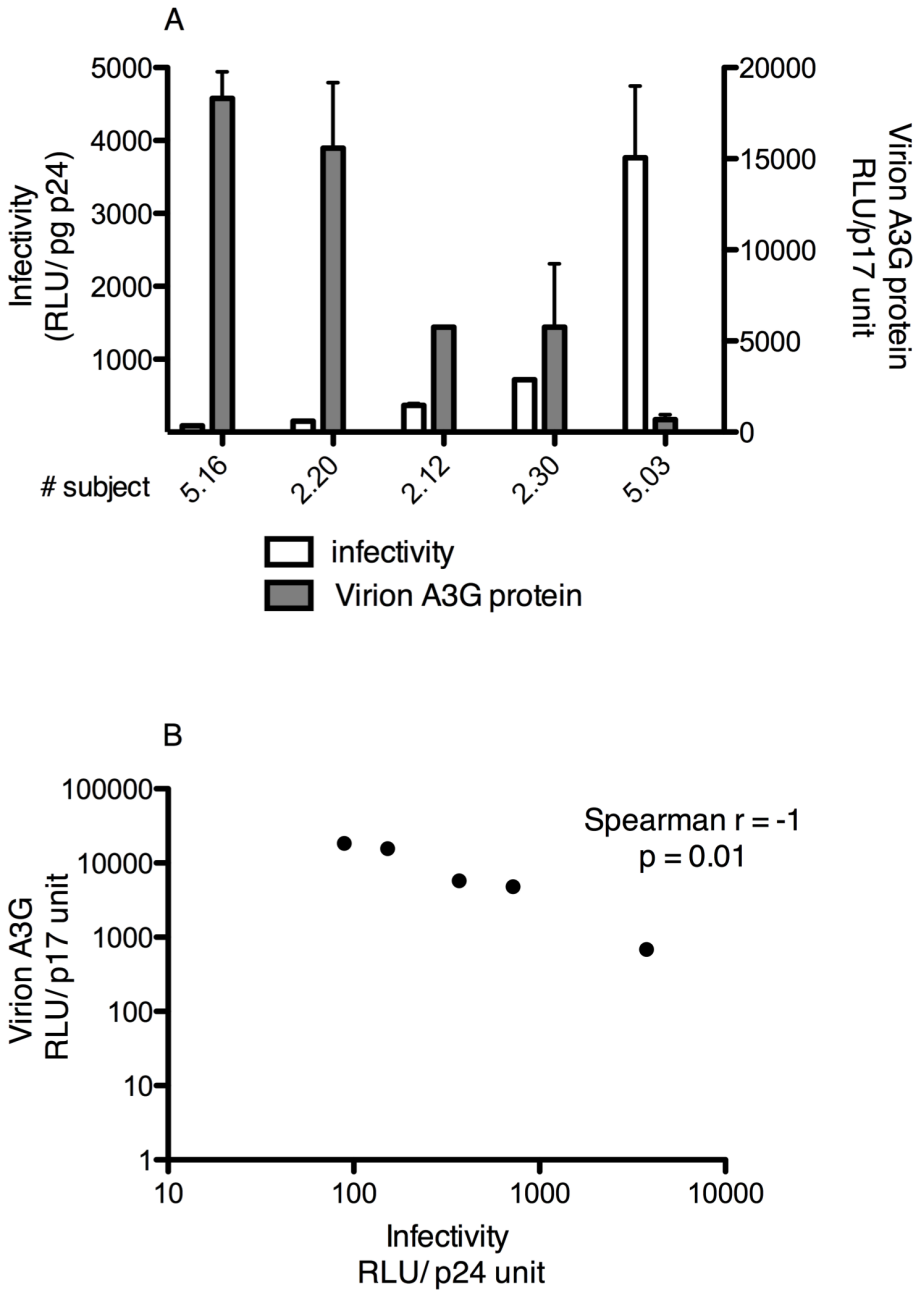
Virion-packaged A3G and infectivity of endogenous viruses produced from activated CD4+ T lymphocytes *ex vivo*. (**A**) Increased virion-packaged A3G protein was associated with decreased infectivity of endogenous viruses produced from activated CD4+ T lymphocytes. Virions released from activated CD4+ T lymphocytes of ART-suppressed (AS) non-controller subjects were analyzed for infectivity using the TZM-bl assay and for virion-associated A3G protein by in-virion Western blotting. CD4+ T lymphocytes were sorted for positivity for CD25, CD69, CD38 and HLA-DR from blood of 5 of the AS non-controller subjects. Activation was maintained in *ex vivo* cultures by anti-CD3/CD28 antibody coated beads. HIV-1 p24 antigen measurement monitored production of endogenous Vif-positive HIV-1 *ex vivo*. Virion content was normalized by p24 antigen amount for both infectivity and virion A3G protein measurements. Bars represent mean +/− standard deviation (SD). (**B**) Virion infectivity was significantly inversely correlated with amount of A3G protein in virions. P value was determined by Spearman correlation.

One explanation for the inverse association between A3G and provirus levels in resting memory cells here, and in the earlier analysis of PBMC [5] could be that Vif-positive virions produced from controllers' cells have more A3G antiviral activity. Therefore, we tested whether higher levels of A3G protein in virions produced from endogenous viruses present in activated primary T cells from HIV-infected subjects led to greater decreases in infectivity and spread of those virions in cultured cells. *In vivo* activated CD4+T cells, defined as positive for CD25, CD69, CD38 and HLA-DR, were sorted from 5 of the AS subjects' PBMCs. Activation was maintained *ex vivo* by adding anti-CD3/CD28 antibody coated beads to cultures. HIV-1 p24 antigen measurements monitored production of endogenous Vif-positive HIV-1. Virion content was normalized by p24 antigen amount for both infectivity and virion A3G protein measurements. Infectivity of endogenous virus in culture supernatant fluids was quantified by TZM-bl assay. Quantitative detection of virion A3G was performed after magnetic bead captured virions were immobilized, fixed, and permeabilized in wells of a 96 well plate. Endogenous Vif-positive virus with lower levels of virion-associated A3G protein had greater infectivity (Fig. 3A). Virion infectivity and A3G content were strongly and significantly inversely correlated (Fig 3B; Spearman r = −1, p = 0.01).

### Decreased spread of HIV-1 virions produced from Tem cells containing higher A3G than Tcm cells

**Figure 4:**
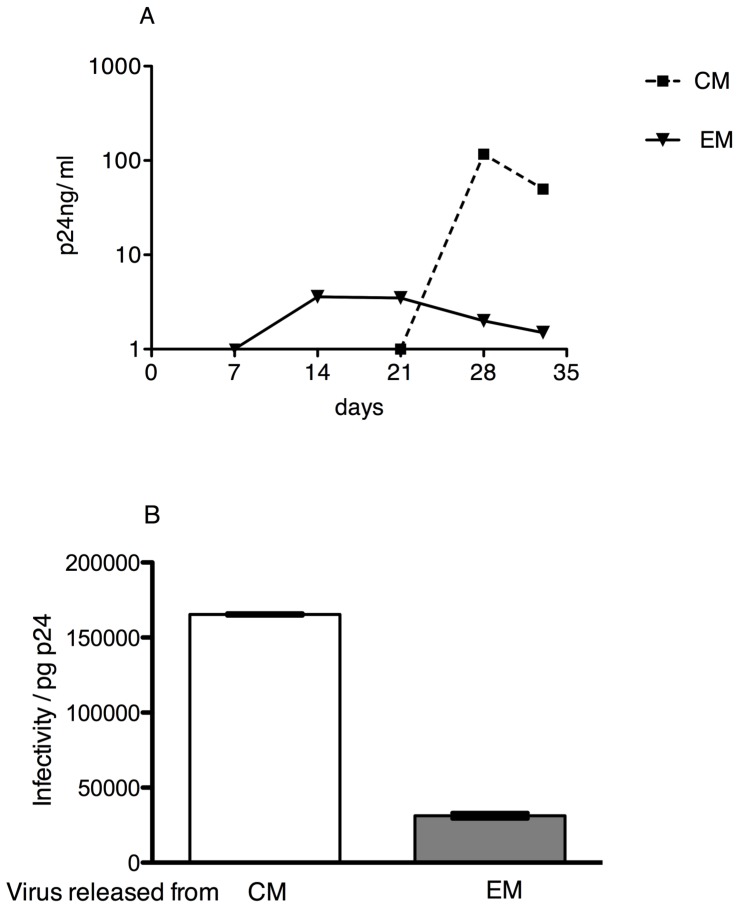
Higher cellular A3G protein levels were associated with decreased infectivity and spread of HIV virions after *ex vivo* activation and infection of primary memory T lymphocytes. (**A**) Blood resting CD4+ T central memory (Tcm) and effector memory (Tem) cells sorted from the same uninfected subject were activated and then infected *ex vivo* with a Vif-positive, CCR5-tropic clinical isolate of HIV. HIV p24 antigen was monitored over time in supernatant fluids from separate Tcm and Tem cell cultures. Virus spread through the culture of Tcm cells more rapidly than it did through the culture of Tem cells previously shown to have higher A3G protein levels (Figure 2). (**B**) Infectivity of virions produced from Tcm cells was greater than that of virions produced from Tem cells. Infectivity was quantified by luciferase activity normalized by amount of p24 antigen added to TZM-bl reporter cells. Bars represent mean +/− standard deviation (SD).

We also evaluated if greater cellular A3G caused increased virion A3G that could decrease Vif-positive HIV-1 spread. We took advantage of the finding that cellular A3G RNA and protein levels were higher in primary Tem than Tcm cells (Fig. 2). Resting Tem and Tcm were isolated from an uninfected subject and activated *ex vivo* with anti-CD3/CD28 antibody coated beads. A3G RNA levels in both Tem and Tcm cells increased over 5 days after this stimulation, such that A3G RNA remained consistently higher in activated Tem than Tcm cells. Activated Tem and Tcm cell cultures were each infected at day 3 after *ex vivo* activation with a replication-competent, CCR5-tropic clinical isolate (MOI of 0.01). This isolate had previously been expanded by passage, and titered, in primary PBMC from uninfected persons, indicating that it carried a functional *vif* gene product. HIV-1 p24 antigen was detected at a low level at day 14 in the Tem culture supernatant fluids, but the levels did not increase after day 14 and declined after day 28 (Fig. 4A). In contrast, HIV-1 p24 antigen levels rapidly and exponentially increased after first detection in supernatant fluids of the Tcm culture, indicating more robust spread through the Tcm culture (Fig 4A). At day 28 of culture, the levels of A3G protein, normalized to levels of actin, were 1.8-fold higher in the Tem cells than the Tcm cells. Virions from day 28 Tem cell culture supernatant fluids contained more A3G than did Tcm-produced virions, after normalization by p24 antigen amount. Virus-containing supernatant fluids from day 28 cultures of activated and exogenously infected Tcm cells had 5.3-fold greater infectivity, normalized for p24 antigen, than those from the parallel Tem cell culture (Fig. 4B). Higher cellular A3G levels increased Vif-positive virion A3G and decreased infectivity of those virions.

## Discussion

Resting CD4+ Tcm and Tem lymphocytes from HLA B57- and/or B27-positive controllers had decreased levels of HIV provirus, compared to cells from AS non-controllers. This confirms earlier reports of decreased PBMC provirus levels in controllers [4], [5], and contrasts with the different results in one report [2]. It also extends analyses from PBMCs to the major latent cellular reservoir in blood of HIV-1 infected individuals with high CD4+ T cell counts and minimal lymphocyte proliferation [6]. Other results of the current study indicated that resting memory T cells with the highest A3G protein levels were significantly more likely to harbor the lowest levels of provirus DNA *in vivo* and that Vif-positive virions containing more A3G had decreased infectivity *ex vivo*. The latter two findings suggest the hypothesis that increased A3G-mediated intrinsic immunity decreases the latent provirus reservoir as one of the multiple mechanisms important for the durability of HIV control in addition to HLA B57- and/or B27-restricted recognition of conserved HIV epitopes by CD8+ CTL. Each of the following associations noted here will be discussed in turn to suggest how future research may further illuminate them: the association of HIV control with decreased provirus, the association of higher cellular A3G protein with HLA B57- and/or B27-associated control, and the inverse association between resting lymphocyte provirus and A3G protein levels.

The association of decreased provirus level with control was seen among VC with consistently low (<2000 copies/ml) viremia in this comparison to AS non-controllers. Therefore, neither undetectable viremia in controllers (e.g.; elite control), nor lack of ART in non-controllers, were required to observe this association. This is not consistent with decreased provirus simply being a by-product of viremia suppression. Data from animal models and recent clinical studies suggest that a decreased provirus reservoir in Tcm can itself slow retrovirus pathogenesis. A decreased reservoir of sooty mangabey SIV (SIVsm) provirus in Tcm is the leading hypothesis to explain the lack of immunodeficiency progression despite sustained, high levels of viremia in infected sooty mangabeys [27]–[29]. In addition, a markedly limited HIV-1 provirus reservoir in Tcm was seen in subjects without favorable HLA alleles and high initial levels of viremia who were started on ART very early after acquiring HIV (in Fiebig stage I) [30]. It has been speculated that viremia-suppressing ART started very early after infection may have minimized expansion of the provirus reservoir in resting Tcm cells of post-treatment controllers to lead to the minimal viremia rebound from such reservoirs following cessation of ART that distinguishes such “post-treatment controllers” from non-controllers [31], [32].

Immune control of HIV in HLA B57- and/or B27-positive subjects was associated here with higher resting lymphocyte A3G protein levels, although a mechanism underlying increased A3G levels was not determined. No human genomic variation has been associated with increased cellular A3G. We hypothesize that better control of Vif-positive HIV replication by HLA-restricted CTL led to preservation of cellular A3G protein levels, because less Vif was produced to deplete A3G. Future studies will be needed to determine whether HLA allele-associated, CTL-mediated control of viremia (or ART) prevents depletion of A3G in controllers' cells during very early infection. It is biologically and clinically important to determine if cellular A3G changes over time after HIV is acquired to test this hypothesis, because it may add support for immediate ART to attempt minimizing Vif-mediated A3G depletion in all HIV-infected subjects.

Whatever the mechanism for increased cellular A3G in HLA B57- and/or 27-positive controllers, higher A3G may add to the multiplicity of factors that slow pathogenesis and extend durability of Vif-positive HIV control. Two different ways in which higher cellular A3G may augment CTL activity have been demonstrated experimentally to date, via either enhanced recognition of HIV-specific antigens encoded by replication-defective, hypermutated proviruses [13] or increased recognition of A3G itself presented on the cell surface (because of greater proteosomal degradation of A3G in Vif-positive HIV-infected lymphocytes) [14]. However, neither report evaluated cells expressing HLA B57 or B27, so relevance for the subjects studied here is uncertain [13], [14]. A3G also has been reported to enhance recognition of HIV-infected T lymphocytes by natural killer (NK) cells [33], although not yet documented *ex vivo* in the one study of this in controllers to date [34]. Non-immune effects of A3G are also possible. Physiologically or pharmacologically increasing cellular A3G levels has augmented its virion packaging and partial antiviral activity, even in the presence of Vif [16], [35]–[43]. Vif antagonism of A3G varies across different HIV clades and virus isolates [44]–[48], suggesting that some Vif-positive HIVs may be more affected by A3G antiviral activities than others. Our earlier report found that greater A3G-mediated hypermutation in controllers' PBMC HIV DNA was associated with lower viremia level [5], and another group has subsequently confirmed that controllers' PBMCs have more hypermutated HIV DNA sequences than do cells from non-controllers [49]. This suggests that greater A3G deaminase activity may provide a larger pool of genetic variants from which mutants that escape from CTL recognition of HLA B57- and/or B27-restricted conserved epitopes might be selected [35], [46], [47], [50]–[52]. The decreased replicative fitness conferred by CTL escape mutations in conserved HIV Gag residues has been hypothesized to be an important factor sustaining HIV control after loss of HLA B57- and/or 27-restricted CTL recognition [15]. Similar complex interactions between innate and adaptive immunity that may sustain control via host-beneficial selection pressures have also recently been suggested for human TRIM5α in HLA B57- and 27-positive controllers [53].

In our earlier work, decreased PBMC provirus level was associated with increased A3G mRNA, but not with hypermutation [5]. This suggested that deaminase-independent mechanisms of A3G may be more relevant than deamination to the decreased provirus levels in controllers' cells. Deaminase-independent interference of virion A3G with reverse transcription (RT) and/or integration [54]–[57] could decrease provirus formation in, and spread to, new target cells; these possibilities are consistent with the current *ex vivo* results showing that increased cellular A3G decreased virion spread through cultured cells.

Cell-intrinsic antiviral effects have also been noted previously in some studies of cells from controllers *in vivo* [4] and *ex vivo* [3], [11], [12]. Decreased RT and integration of HIV-1 were seen in resting CD4+ T cells from controllers relative to those from uninfected subjects after *ex vivo* infection with replication-competent, Vif-positive laboratory strains of HIV-1 without spinoculation or activation [3]. It was not seen with spinoculation [2]. The mechanism for the observed block to integration *ex vivo* in cells from controllers was not defined, although p21 was implicated in decreasing RT [3], [11]. Increased target cell A3G [57] provides a candidate mechanism that can be studied in the future to determine if it may account for the decreased integration in controllers' cells *ex vivo* [3], [11], [12].

The highest levels of A3G protein in resting memory CD4+ T cells were significantly associated with the lowest levels of provirus DNA, and significantly more common among the controller than non-controller subjects studied here. These hypothesis-generating *in vivo* data suggested that more A3G in Vif-positive viruses better blocked provirus formation in the next target cell (e.g.; decreased virus spread). The alternate explanation for the association between decreased provirus and higher A3G is that increased provirus in non-controller cells led to a greater decrease in lymphocyte A3G levels. The earlier finding of this association in PBMC from VC versus untreated non-controller subjects [5] did not exclude the possibility that more HIV-1 Vif expressed during active HIV replication in untreated non-controllers' cells increased A3G's proteosomal degradation. There are two reasons why increased Vif degradation of A3G seems less likely as an explanation for the association being observed in the current study. All subjects in this current study had low viral load (median 57 copies/ml), as well as high CD4 cell counts (median 707 cells/ml). This was planned to allow similar low levels of productively-infected cells expressing HIV-1-Vif in blood from both subject groups. Moreover, a second reason is that resting Tcm cells studied here do not support active HIV-1 replication needed for *Vif* expression that would lead to A3G degradation. Additional experiments also directly tested whether increased producer cell A3G decreased Vif-positive HIV replication *ex vivo*. Using two different experimental approaches, we found that Vif-positive virions containing relatively higher levels of A3G had more decreased virion infectivity. Cells with higher A3G produced virions with more A3G packaged within them. These *ex vivo* results are consistent with A3G causing decreased provirus, but further work is needed to conclusively support this explanation of the association observed here. There were not adequate numbers of cells from these subjects to attempt *ex vivo* manipulations to specifically decrease A3G in controller cells, or increase A3G in non-controller cells. Such studies could more definitively establish causation. *In vivo* testing of the causation hypothesis will require a clinical intervention that specifically affects A3G's anti-HIV activity; this is not yet available. Additional hypotheses concerning the incremental virion-packaged A3G resulting from increased cellular A3G are also of interest for future investigation, including where the increased A3G is localized within the virion and whether its anti-HIV activity in virions is deaminase-dependent.

Further characterization of how provirus burden is limited in controllers' cells could help reach the goal of functionally curing HIV-1 infection. Whenever ART is stopped in a non-controller who did not start ART in the first weeks after acquiring HIV, previously quiescent HIV-1 proviruses reactivate from blood memory cells to cause rebound viremia [58], [59]. Strategies are now under study to attempt a functional cure of HIV-1 that will enable post-treatment control, as has occurred in only a few to date after stopping ART [31], [32], [60], [61]. In addition to very early treatment initiation, a promising approach involves development of “latency reversing agents” to reactivate transcription of latent proviruses in resting memory lymphocytes [62]. Enhancement of immune-mediated clearance of cells producing reactivated HIV-1 may also be needed [63], and it is not yet clear if ART will completely prevent spread of virions reactivated from memory CD4+ T cells. The current study suggests that enhancing the relatively low levels of resting memory lymphocyte A3G may increase its packaging into reactivated virions and limit reactivated virion spread to new targets. Much could be learned from including controllers with higher cellular A3G levels in trials of latency reversing agents. This study also highlights the potential of identifying novel biologicals or small molecules that specifically enhance cellular A3G levels or activity [36]–[43]. An A3G-booster could help rigorously establish one or more causal mechanisms for A3G in HIV control *in vivo*, and perhaps aid efforts to functionally cure HIV-infected subjects as a temporary adjunct to ART when latency reversing agents are used.

## Supporting Information

Figure S1 **A3G RNA expression** ***in vivo*** **in CD4+ T lymphocytes of viremic controller (VC) subjects.** CD4+ T lymphocytes were sorted as described in Methods into resting naïve (naive), resting central memory (CM), resting effector memory (EM), and activated cells (AC). A3G RNA levels were determined by qRT-PCR. Lines represent median values. P values were determined by 2-tailed Mann-Whitney test.
(TIF)

Figure S2 **A3G RNA and protein levels increase after** ***ex vivo*** **activation of primary CD4+ T lymphocytes.** (**A**) A3G RNA and protein both increase after *ex vivo* stimulation of the T cell receptor (TCR) of primary CD4+ T lymphocytes of infected subjects by anti-CD3/CD28 beads. RNA levels were determined by qRT-PCR. A3G protein levels were determined by quantitative immunoblotting and normalized to β-actin. (**B**) Immunoblots of A3G protein, and actin loading control, from a representative infected subject from day 0 to day 5 after anti-CD3/CD28 bead stimulation of TCR. Increasing A3G (lower band) is evident at day 3 and 5, while actin (upper band) remains relatively unchanged after anti-CD3/CD28 bead stimulation.
(TIF)

Figure S3 **Percentages of activated (A) and resting memory (B) cells among CD4+ T lymphocytes** ***in vivo*** **.** (**A**) A higher percentage of *in vivo* activated cells are present in blood CD4+ T lymphocytes from antiretroviral-suppressed (AS) non-controllers than either viremic controllers (VC) or HIV-negative subjects. CD4+ T cells were enriched by negative selection. Activated cells were defined as expressing HLA-DR, CD25, CD69, and CD38 by flow cytometry. (**B**) Comparison of percentages of resting CD4+ T memory cells in CD4+ T cells from blood of VC, AS non-controller, and uninfected subjects. No differences were found in percentages of Tcm in VC, AS non-controller, and uninfected subjects. Flow cytometry categorized resting CD4+ central memory T cells as CD45RO+, CCR7+ and resting T effector memory cells as CD45RO+, CCR7-. In both (**A**) and (**B**), percentages are relative to total number of CD4+ T cells. Lines in plots represent median and inter-quartile range (IQR) values. P values were determined by 2-tailed Mann-Whitney test. Only p values that reached statistical significance are indicated.
(TIF)
