# Supplementary figures and images for: Lower HIV Provirus Levels Are Associated with More APOBEC3G Protein in Blood Resting Memory CD4+ T Lymphocytes of Controllers In Vivo
Source: PLoS One. 2013 Oct 16;8(10):e76002. doi: 10.1371/journal.pone.0076002 (PMC3797809; doi:10.1371/journal.pone.0076002)

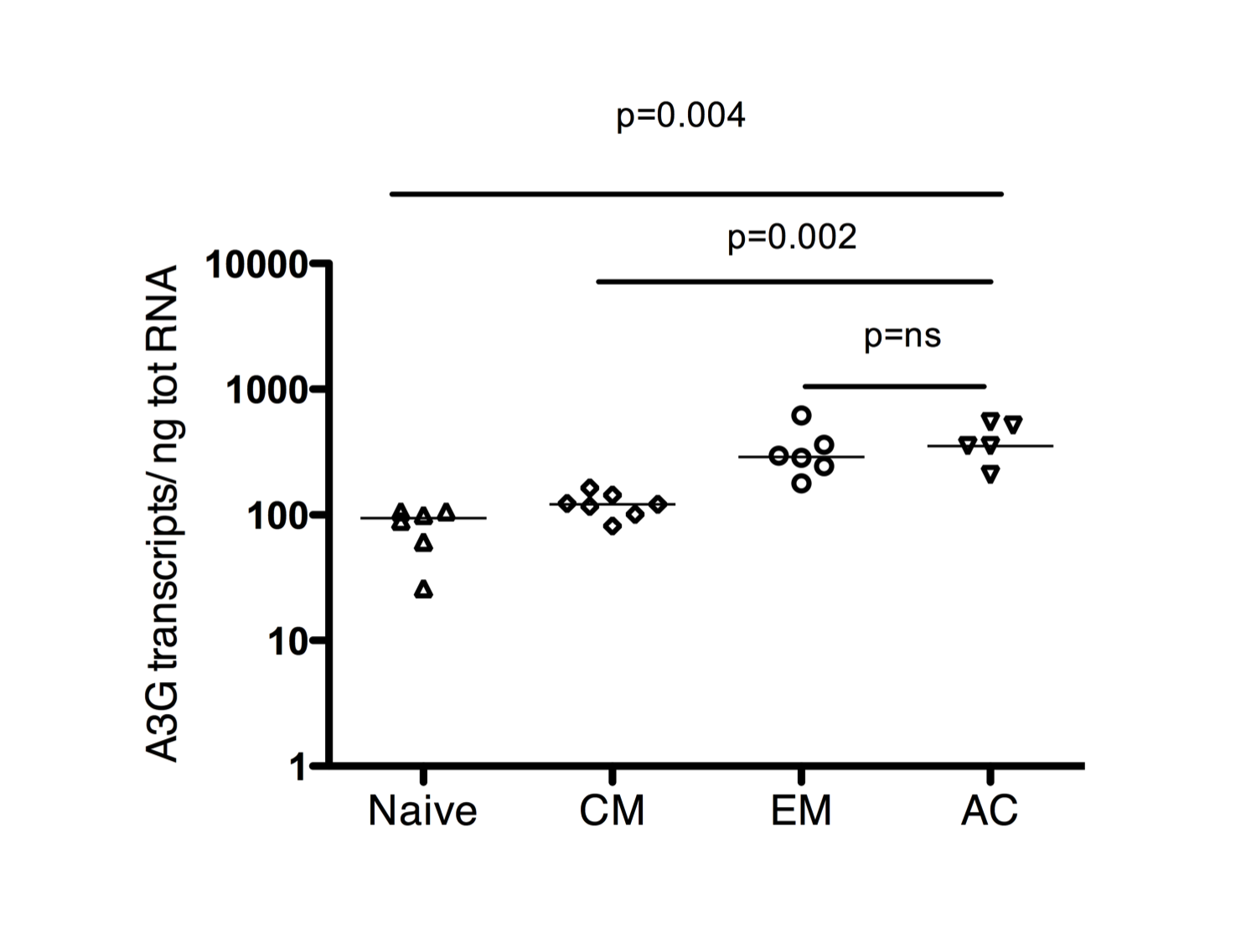

Supplement: Figure S1 — A3G RNA expression in vivo in CD4+ T lymphocytes of viremic controller (VC) subjects. CD4+ T lymphocytes were sorted as described in Methods into resting naïve (naive), resting central memory (CM), resting effector memory (EM), and activated cells (AC). A3G RNA levels were determined by qRT-PCR. Lines represent median values. P values were determined by 2-tailed Mann-Whitney test. (TIF) [file pone.0076002.s001.tif]

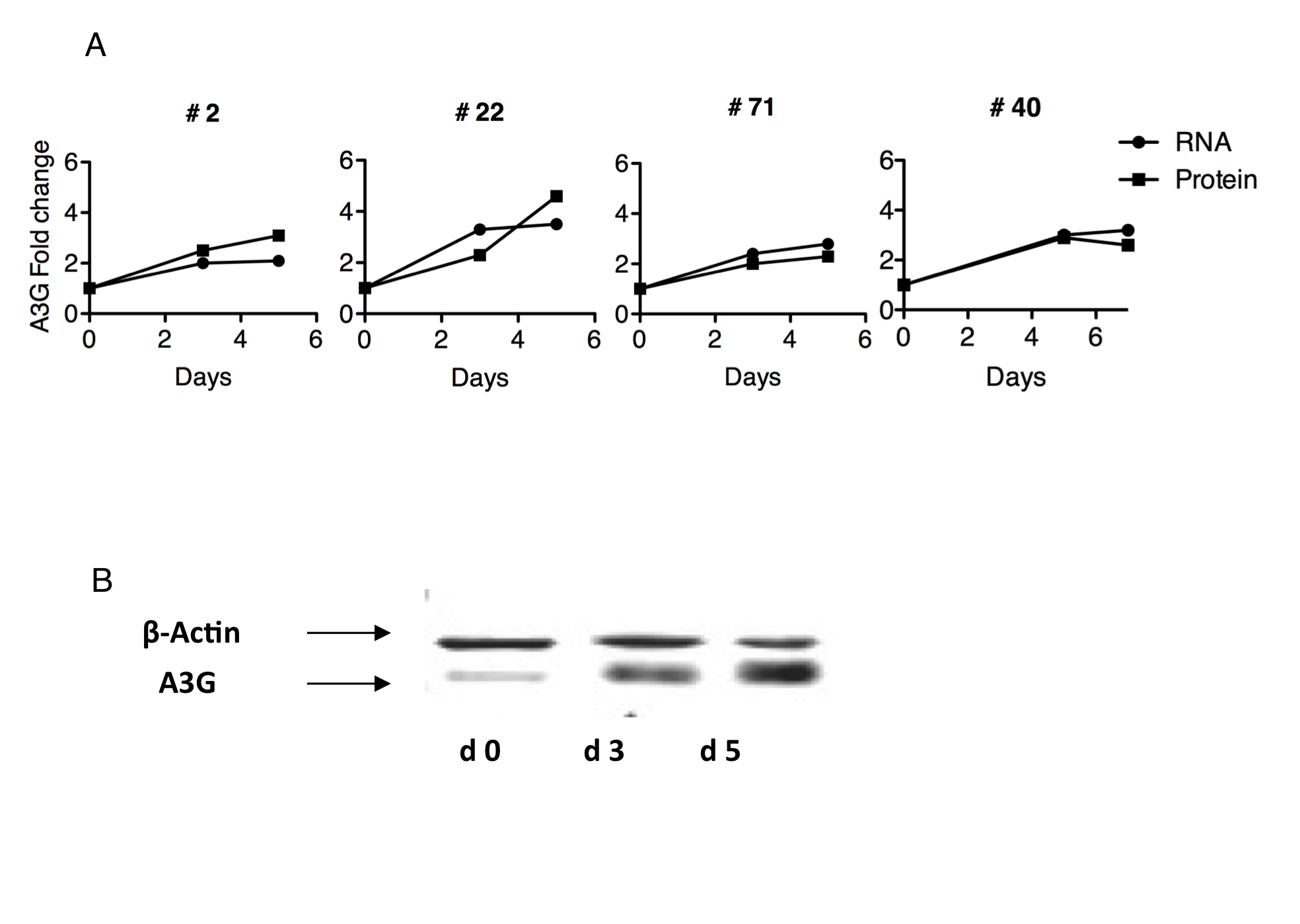

Supplement: Figure S2 — A3G RNA and protein levels increase after ex vivo activation of primary CD4+ T lymphocytes. (A) A3G RNA and protein both increase after ex vivo stimulation of the T cell receptor (TCR) of primary CD4+ T lymphocytes of infected subjects by anti-CD3/CD28 beads. RNA levels were determined by qRT-PCR. A3G protein levels were determined by quantitative immunoblotting and normalized to β-actin. (B) Immunoblots of A3G protein, and actin loading control, from a representative infected subject from day 0 to day 5 after anti-CD3/CD28 bead stimulation of TCR. Increasing A3G (lower band) is evident at day 3 and 5, while actin (upper band) remains relatively unchanged after anti-CD3/CD28 bead stimulation. (TIF) [file pone.0076002.s002.tif]

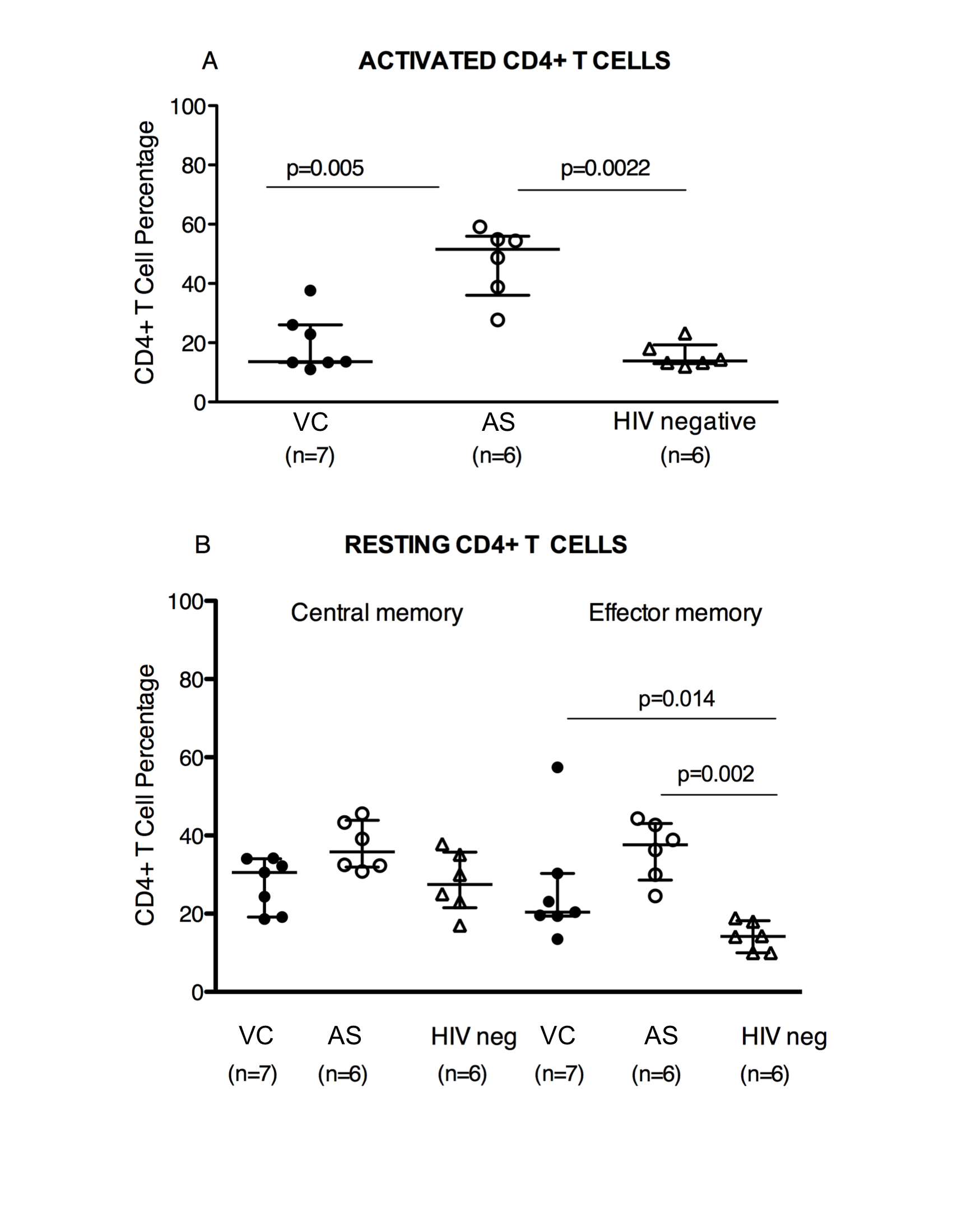

Supplement: Figure S3 — Percentages of activated (A) and resting memory (B) cells among CD4+ T lymphocytes in vivo . (A) A higher percentage of in vivo activated cells are present in blood CD4+ T lymphocytes from antiretroviral-suppressed (AS) non-controllers than either viremic controllers (VC) or HIV-negative subjects. CD4+ T cells were enriched by negative selection. Activated cells were defined as expressing HLA-DR, CD25, CD69, and CD38 by flow cytometry. (B) Comparison of percentages of resting CD4+ T memory cells in CD4+ T cells from blood of VC, AS non-controller, and uninfected subjects. No differences were found in percentages of Tcm in VC, AS non-controller, and uninfected subjects. Flow cytometry categorized resting CD4+ central memory T cells as CD45RO+, CCR7+ and resting T effector memory cells as CD45RO+, CCR7-. In both (A) and (B), percentages are relative to total number of CD4+ T cells. Lines in plots represent median and inter-quartile range (IQR) values. P values were determined by 2-tailed Mann-Whitney test. Only p values that reached statistical significance are indicated. (TIF) [file pone.0076002.s003.tif]
